# Large Scale Identification and Categorization of Protein Sequences Using Structured Logistic Regression

**DOI:** 10.1371/journal.pone.0085139

**Published:** 2014-01-20

**Authors:** Bjørn P. Pedersen, Georgiana Ifrim, Poul Liboriussen, Kristian B. Axelsen, Michael G. Palmgren, Poul Nissen, Carsten Wiuf, Christian N. S. Pedersen

**Affiliations:** 1 Centre for Membrane Pumps in Cells and Disease - PUMPKIN, Danish National Research Foundation, Aarhus C, Denmark; 2 Department of Molecular Biology, Aarhus University, Aarhus C, Denmark; 3 Bioinformatics Research Centre, Aarhus University, Aarhus C, Denmark; 4 Swiss-Prot Group, Swiss Institute of Bioinformatics, CMU, Geneva, Switzerland; 5 Department of Plant and Environmental Sciences, University of Copenhagen, Frederiksberg C, Denmark; 6 Department of Mathematical Sciences, University of Copenhagen, Copenhagen Ø, Denmark; 7 INSIGHT Centre for Data Analytics, University College Dublin, Dublin, Ireland; International Centre for Genetic Engineering and Biotechnology (ICGEB), India

## Abstract

**Background:**

Structured Logistic Regression (SLR) is a newly developed machine learning tool first proposed in the context of text categorization. Current availability of extensive protein sequence databases calls for an automated method to reliably classify sequences and SLR seems well-suited for this task. The classification of P-type ATPases, a large family of ATP-driven membrane pumps transporting essential cations, was selected as a test-case that would generate important biological information as well as provide a proof-of-concept for the application of SLR to a large scale bioinformatics problem.

**Results:**

Using SLR, we have built classifiers to identify and automatically categorize P-type ATPases into one of 11 pre-defined classes. The SLR-classifiers are compared to a Hidden Markov Model approach and shown to be highly accurate and scalable. Representing the bulk of currently known sequences, we analysed 9.3 million sequences in the UniProtKB and attempted to classify a large number of P-type ATPases. To examine the distribution of pumps on organisms, we also applied SLR to 1,123 complete genomes from the Entrez genome database. Finally, we analysed the predicted membrane topology of the identified P-type ATPases.

**Conclusions:**

Using the SLR-based classification tool we are able to run a large scale study of P-type ATPases. This study provides proof-of-concept for the application of SLR to a bioinformatics problem and the analysis of P-type ATPases pinpoints new and interesting targets for further biochemical characterization and structural analysis.

## Introduction

Systematic sequencing efforts in the last decade have provided complete sequences of an increasing number of genomes, and a large amount of sequence information is available from other organisms. A traditional analysis based on a multiple sequence alignment (MSA) and tree reconstruction might be computational feasible for up to ∼100k sequences using fast MSA heuristics such as MAFFT and efficient implementations of the canonical neighbour-joining (NJ) method such as QuickTree [30] or RapidNJ [47], or heuristics such as ClearCut [46]. For larger-scale sequence classification, machine learning based methods such as (profile) hidden Markov models (HMM) and Support Vector Machines (SVM) are applicable. These machine learning methods are trained on a subset of the data and then used to rapidly classify unknown sequences.

A possible alternative to HMM and SVM is Structured Logistic Regression (SLR) [23]. SLR is a recently developed machine learning method that has not been previously applied to large-scale classification problems in bioinformatics, but have shown great promise in other types of classification [23]. In this paper we provide a proof-of-concept application of SLR to a large-scale classification problem in bioinformatics. We use classification of P-Type ATPases as our application because we believe it can generate important biological information. Also the rapidly increasing number of possible P-type ATPases calls for an automated procedure to facilitate the quick analysis of their distribution into different classes to guide biochemical experiments. Since SLR has been shown previously to compare favourable with SVM [23], we have chosen to compare the performance of our SLR based classifier to an profile HMM based classifier, and, for a smaller set of sequences, to a traditional MSA-NJ analysis. A variant of SLR has been developed recently and it has been validated experimentally on biological sequence classification, where it performed favourably [49]. However, it focused on extending the learning framework, rather than the key biological insights made accessible by SLR, and the scale of the performed experiments was much smaller (∼150.000 sequences) as compared to this paper (∼10 million sequences).

P-type ATPases are a family of proteins involved in the active pumping of charged substrates across biological membranes [1]. Their distinguishing feature is the formation of a phosphoaspartate intermediate formed at a canonical DKTGT sequence motif (hence P-type) [2], [3]. P-type ATPases of various substrate specificities have several vital cellular functions. For example, they provide the basis for action potentials in nervous tissues, secretion and re-absorption of solutes in the kidneys, acidification of the stomach, Ca^2+^-dependent signal transduction, and lipid bilayer asymmetry. X-ray structures of three types of P-type ATPases exist [4], [5], [6] leading to a very detailed mechanistic understanding of their general function [7], [8].

Several reports have speculated about the relationship among the various P-type ATPases [9], [10], [11], [12], [13], but the number of sequences included in these studies has been relatively low. A breakthrough in the classification was made in 1998 by Axelsen & Palmgren, leading to a clear conceptualization of the different classes found in this family. Since then, one new subclass of P-type ATPases has been suggested [14] as well as a completely revised classification-scheme [12], [13].

Based on sequence homology, the P-type ATPase family is divided in 5 superclasses (I to V) and further into 11 different subfamilies or classes [11], [14]. Each class appears specific for a particular type of substrate (Table 1). Class IA is part of a large protein complex called KdpABCF involved with K^+^ uptake. Class IB groups the heavy-metal ATPases, including copper and zinc exporters. Class IIA includes the Ca^2+^ and Mn^2+^ ATPases [15], [16], [17]. Class IIB contains primarily calmodulin-binding Ca^2+^-ATPases. Class IIC consists of the Na^+^/K^+^ and H^+^/K^+^ ATPases, while class IID is involved in transport of Na^+^ and K^+^ [18], [19]. Class IIIA consists of the plasa membrane H^+^ P-type ATPases [20] and class IIIB of the Mg^2+^ importers [21]. Class IV groups proposed phospolipid translocases [22], while classes VA and VB contain ATPases with unknown specificity located in the endoplasmatic reticulum membranes of eukaryotes [14].

**Table 1:** Overview of the canonical P-type ATPase classes.

Class	Substrate out	Substrate in	Expected TMs ^(a,b)^	Taxonomic coverage ^(b)^
IA	unknown ^(c)^	K^+^	7	Prokaryotic
IB	HM^ (d)^	unknown	6–8	All kingdoms
IIA	Ca^2+^ or Mn^2+^	H^+^	10	All kingdoms
IIB	Ca^2+^	H^+^	10	All kingdoms
IIC	Na^+^ or H^+^	K^+^	10	Metazoa
IID	Na^+^ or K^+^	unknown	10	Fungi
IIIA	H^+^	none ^(e)^	10	All excl. Metazoa
IIIB	unknown	Mg^2+ (f)^	10	Prokaryotic
IV	unknown	PL ^(g)^	10	Eukaryotic
VA	unknown	unknown	12	Eukaryotic
VB	unknown	unknown	12	Eukaryotic

a) TM: transmembrane helices. b) Expected from the literature. See main text for references. c) Substrate have not been identified. d) HM: heavy metals. Primarily Cu and Zn, but also Co, Cd, Ag and Pb. e) Possibly no countertransport. f) Transported with the electrochemical gradient. g) PL: Phospholipids.

We have applied SLR-classifiers to the entire UniProtKB v. 15.8 [24] to identify new P-type ATPases and further classify them into the 11 known subfamilies. To examine the per-species distribution of ATPases, we have analyzed 1,123 genomes. Furthermore, an analysis of the predicted membrane topology of P-type ATPases found in these genomes shows that the transmembrane region can be described as a three component system containing a core region of 6 transmembrane helices and two elements that reside on the N- and C-terminal part.

## Methods

### Description of Structured Logistic Regression

Structured Logistic Regression (SLR) is a machine learning tool first proposed in the context of text categorization [23]. SLR takes as input a training set of n samples, {*x_i_*, *y_i_*}, *i* = 1,..., *n*, where *x_i_* is a sequence, and *y_i_* ∈ {+1,−1} are labels indicating the class. The SLR output is a set of discriminating subsequences of unrestricted length (also known as *k*-mers or *n*-grams, with *k* or *n* unrestricted in this case; in this work we refer to them simply as predictors) together with their weights *w_j_* indicative of their discriminative power. The SLR decision function is linear: 
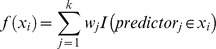
where *k* is the total number of selected predictors and I(.) is the indicator function. To predict class membership of *x_i_*, the score *f* (*x_i_*) is related to the probability that *x_i_* belongs to class +1:
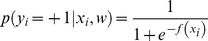



The learning algorithm is based on a coordinate-wise gradient ascent optimization technique for iteratively maximizing the likelihood of the training set [23]. Upon optimizing the likelihood, the algorithm outputs a compact set of discriminative predictors to be used for classification. The output can thus be analysed in order to understand what lead to a certain classification decision, i.e., the user can simply go over the list of positive and negative predictors to study the characteristic subsequences for each of the predefined subfamilies. SLR lets the data drive the predictor selection process, without assumptions on the underlying data distribution or constraining the predictors according to manually built regular expression rules. Further details on this method can be found in [25].

### Datasets

#### Positive dataset

For positive training examples, we built a P-type ATPase dataset consisting of 397 sequences from PAT-base (http://www.traplabs.dk/patbase) and 93 sequences from the dataset of Møller et al., resulting in 490 ATPases [11], [14].

#### Negative dataset

For negative training examples, we selected all sequences from the *Homo sapiens* genome (eukaryota), the *Escherichia coli* genome (bacteria) and the *Thermoplasma acidophilum* genome (archaea). About 30 of these sequences contained the motif DKTGT considered to be characteristic of P-type ATPases. We manually inspected this small set of sequences and confirmed that they had all previously been reported as P-Type ATPases in these genomes (cf. PAT-base). We removed these sequences, giving us a total of 43.315 sequences in the negative dataset. The use of complete genomes from different life domains ensured that all types of protein sequences were represented in the negative training set.

#### UniProtKB dataset

To identify new ATPases we used the UniProtKB v15.8 (downloaded Oct. 2009) containing the bulk of known protein sequences (a total of 9,325,547 sequences).

#### Genome dataset

To analyse the organismal distribution of P-type ATPases, we used translated protein sequences from 1,123 eukaryotic, bacterial and archaeal genomes publicly available from Entrez (downloaded Feb. 2010 from http://www.ncbi.nlm.nih.gov/sites/genome), a total of 4,131,203 protein sequences. These include different isoforms from alternative splicing of eukaryotic genes. Genomes from different strains of the same species were included, but analysed separately (e.g., *E. coli* is represented in the dataset with 29 different strains). Furthermore the data was merged on organism level with only one copy of each unique chromosome. As an example, the array of human sequences in our dataset consists of translated protein sequences from 25 chromosomes (22 autosomes, 2 gonosomes and 1 mitochondrial chromosome).

### Checking the quality of the positive dataset

The quality of any machine learning tool depends heavily on the quality of the positive dataset used for training, thus we initially checked how well the 11 classes separated in the positive dataset. The original procedure for classifying the sequences relied on manually selecting a conserved sequence kernel with 8 elements from each sequence, aligning them and generating a neighbour-joining tree [11], [14]. This is a highly successful approach, but ultimately subjective and non-scalable to larger datasets. Using full sequences, we generated an iterative multiple sequence alignment and a bootstrapped minimum evolution tree to check if the 11 subfamilies are indeed distinguishable. This is a very powerful method of grouping sequences, but only feasible for small datasets due to prohibitive computational costs. We observe the same results as Axelsen & Palmgren with some possible sub-branching of class IIA (Figure S1 in File S1), emphasising that the initial classification observed by them is consistent with newer classification methods and that the quality of the positive dataset is good.

### Classification tasks

#### Task 1: Identifying P-type ATPases

This task focuses on predicting whether a sequence is a P-type ATPase or not using SLR. As training data we used the positive and the negative datasets described in Section 2.2, a total of 43,805 sequences (490 positive and 43,315 negative). Training SLR on this dataset took under 1 minute. This classifier was then applied to the UniProtKB and Genome datasets to identify new P-Type ATPases.

#### Task 2: Fine-grained classification of P-type ATPases

**Figure 1:**
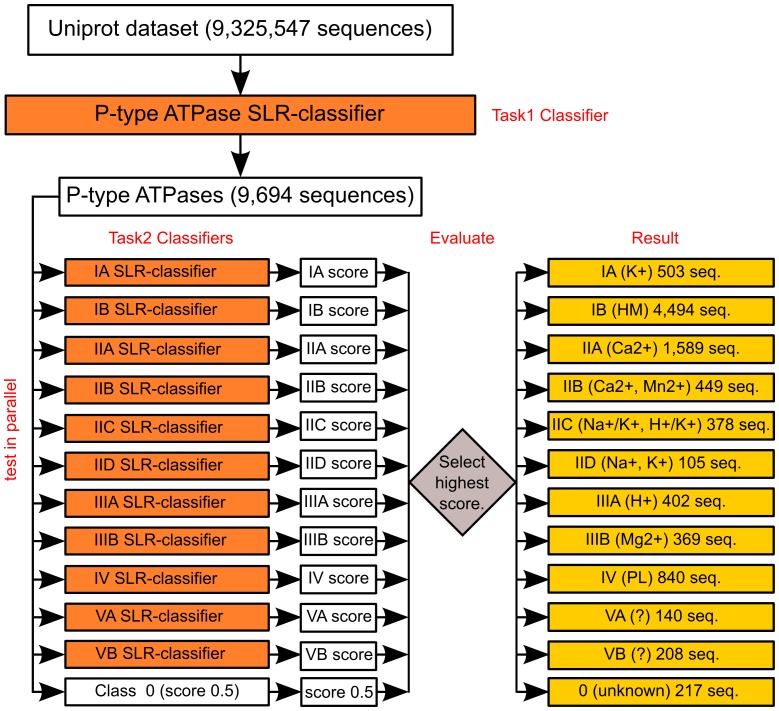
Flowchart of the SLR classification approach. Classification is based on 12 SLR-classifiers (orange). The numbers noted in parenthesis are the classification-result using the UniProtKB dataset as an example. HM: heavy metals. PL: Phospholipids.

The second task focuses on organizing the P-type ATPases identified in Task 1 into the 11 known classes. Using the positive dataset for training (490 P-type ATPases classified into 11 classes) we applied a one-versus-all approach for building binary training sets for each class. In this framework, each sequence gets a classification score for each of the classes tested. SLR outputs the probability of a target sequence to belong to the positive class. For each sequence, we rank the 11 scores and classify the sequence to the maximum probability class. Sequences with a probability less than 0.5 for all 11 classes are collected into a group called class 0 with assigned score of 0.5. We store all 11 scores for each sequence in the database to preserve information on how far a sequence was from inclusion in any of the 11 classes. We base our initial training of the Task 2 classifiers on the 490 sequences in the positive dataset for which the distribution into subclasses are known. Since this set is fairly small (only 490 sequences split into positives and negatives) compared to the number of P-Type ATPases identified by our Task 1 classifier, we investigated several *retraining techniques* to improve the initial Task 2 classifiers in an iterative process with the goal of improving the final classification. We observed that the retraining procedure presented in [26] worked best for our classification problem. Namely, we retrained the classifiers until no more test sequences had their labels re-assigned. For example, for the UniProtKB dataset, we started with the training set of 490 ATPases and classified the 9,694 UniProtKB test sequences identified in Task 1. Next, we iteratively used the newly labeled test sequences to re-train the classifiers and re-label the full UniProtKB test set. Since the start classifiers are already quite accurate, this process stops with a stable labeling of the test set and the final classifiers are highly accurate. The classification process and the overall approach are sketched in Figure 1 using the UniProtKB dataset as an example. The final predictors for each class can be seen in Table S3 in File S1. The final classifiers are available as an online tool (http://www.pumpkin.au.dk/pump-classifier/) for the classification of new unknown sequences.

#### Task 3: Curation

The P-Type ATPases identified in Task 1 were manually inspected for obvious false positives, e.g., extremely short or long sequences. P-type ATPases are typically between 600 and 2000 amino acids long. We did not use any sequence length filter prior to applying the SLR classifiers to allow SLR to also identify P-type ATPases sequence fragments. SLR actually classifies most sequence fragments correctly, as validated by alignment to full-length sequences of the similar proteins found by BLAST [44]. False positives obvious to a human, mostly virus envelope sequences with <50 amino acids, as well as sequences from DNA gyrases and dystonins (which contain DKTGT as part of their sequence), were removed. The envelope sequences most likely appear because the negative training dataset did not contain virus sequence data.

### Tools and parameters

#### Comparing SLR to HMM

In order to estimate the classification quality of SLR vs. HMM for Task 1 we have done 10-fold cross validation on the training data (positive and negative dataset). We split the training data (490+43315 sequences) into 10 groups, generated a single HMM or SLR using the sequences from the 9 groups and tested on sequences in the 10th group. This was done 10 times. We used the same test folds for SLR and HMM. The only difference is that HMM uses only positive examples for training, while SLR uses both positive examples and negative examples for training.

For the HMM approach we used HMMER v. 3.0 with default settings [43] and for the SLR approach we used SLR v. 1.0.1 with default settings. Cut-off thresholds were determined by the algorithms, and not manually optimized in either case.

**Parameters for checking the positive dataset.** For the multiple sequence analysis (MSA) we used MUSCLE [27] with default settings and max 32 iterations. We computed a Minimal Evolution Tree using MEGA4, and calculated bootstrap values with 500 trials [28]. Our reason for using MUSCLE and ME is that the size of the positive dataset (490 sequences) makes it computational feasible to use these more precise methods rather than the computationally faster, but potentially less precise, tools MAFFT [29] and QuickTree [30] that we use for the analysis of the much larger UniProtKB dataset (9,694 sequences).

**SLR Training parameters.** We used SLR version 1.0.1. We trained the SLR classifiers using slr_learn and slr_mkmodel with default parameters [25]. We tested the classifiers using the slr_classify software.

**MSA-NJ analysis on UniProtKB.** For multiple sequence analysis of the ATPases identified in the UniProtKB dataset we applied MAFFT [29] with default settings. The large number of sequences (9,694) makes it computational infeasible to use a potential more precise iterative alignment method such as MUSCLE as we did in the validation of the positive dataset. For tree construction we used the neighbour-joining (NJ) algorithm as implemented in Quicktree with default settings [30]. Building an NJ-tree of the 9,694 sequences in the UniProtKB dataset took about 3 hours using QuickTree on a standard Linux machine. Again, the large number of sequences (9,694) in the dataset makes it computational infeasible to use a potential more precise method such as Minimal Evolution as we did in the validation of the positive dataset. To visualize the tree, we used Dendroscope [31].

#### Membrane topology analysis

For the membrane topology analysis we employed Phobius [32]. We found the location of the N- and C-terminal elements by matching the topology-result with the position of the DKTGT motif that is always located after transmembrane helix 4 (M4) of the core [2], [3], [6], [45].

## Results and Discussion

### Structured Logistic Regression for identification of P-type ATPases

The objectives of the present work was to identify all sequences of the P-type ATPase super family currently present in protein databases (Task 1) and subsequently to categorize the identified sequences into subfamilies (Task 2). For this purpose we used Structured Logistic Regression (SLR), which is a machine learning tool first proposed in text categorization. From a training set containing examples sequences from two classes, SLR learns a set of discriminating motifs and associated weights, such that deciding whether a novel sequence belongs to one class or the other can be done by determining the presence of the motifs in the sequence. The total weight of all motifs present (i.e. all motifs that occur in the novel sequence at some position) determines which class the novel sequence belongs to.

Simple methods for extracting P-type ATPase sequences from large datasets are already available [11], which are based on a PROSITE motif covering DKTGTLT (PS00154) and a PFAM profile (PF0122) that is a little less specific, i.e. it includes some false positives. Therefore, by comparing the results of these simple but reliable methods with that of the more sophisticated SLR approach it was possible to evaluate the results of the latter.

As explained in further details below, we have applied our Task 1 SLR-classifier to the ∼9.3 million sequences in the UniProtKB dataset. It identified 9,634 sequences as P-type ATPases of which 22 sequences (Table S4 in File S1) do not contain an intact DKTGT site but a further analysis revealed that they are indeed P-type ATPases. These 22 sequences would not have been identified by PROSITE although they are identified by PFAM. For unequivocal identification of P-type sequences, the results of the SLR method could favorably be filtered to include only those sequences with the slightly longer PROSITE motif (PS00154) covering DKTGTLT (with a few variations), which seems to eliminate all false positives.

### Performance of Structured Logistic Regression vs. Hidden Markov Models for identifying P-type ATPases

We compared the SLR-classifiers generalization ability to that of an HMM approach for Task 1 (identifying P-type ATPases) by 10-fold cross validation. We generated the Receiver Operating Characteristic curve (ROC curve) and calculated the area under the ROC curve (AUC). Here both methods excel (Table 2). As expected HMM works well, and we are encouraged to observe that SLR gives comparable results. Calculating the true positive (TP) and false positive (FP) rate, we observe that both SLR and HMM mainly retrieve true positives, with SLR being slightly more conservative than HMM. However SLR is superior at reducing the false positives with a FP-rate of virtually zero (Table 2). As the number of true positives in the dataset is very small compared to the total number of true negatives, the calculated ratios should be considered with caution. Still, the difference in FP-rate is significant due to the large number of sequences we ultimately want to test. HMM requires some manual work in terms of optimizing thresholds to reduce the FP-rate to an acceptable level.

**Table 2:** Comparison of running time of SLR to HMM for task 1.

Classifier	AUC	% TP	% FP	CPU Running time ^(a)^
SLR	99.61%	99.1901%	0.0000%	19 min
HMM	99.99%	100.0000%	0.3396%	20 min+150 min ^(b)^

a) CPU running time for complete training and classification of UniProtKB.

b) HMM running time is split into time for constructing a MSA and time for actual training/classification.

As a further argument for SLR vs. HMM, its running time compared to HMM for the complete classification of the ∼9.3 million sequences (Table 2) is about 8 times faster than that of HMM.

### Performance of the SLR-classifiers in Task 2

To assess the SLR-classifiers ability to classify ATPases into the 11 classes in Task 2, we ran 1000 experiments per class with random splits into 90% training and 10% test using the positive dataset. SLR is highly accurate at identifying ATPase subfamilies (Table S1 and Table S2 in File S1). The IA, IID and IIIB classifiers are slightly worse than the others. This might be due to a lower number of positive training sequences for these classes compared to other classes, but might also be due to the intrinsic qualities of these classes. Overall, we find that the validated accuracy is very high for this difficult task.

### Running the SLR-classifiers on the UniProtKB dataset

Being satisfied that SLR is appropriate for the classification task, we applied the SLR-classifiers to the ∼9.3 million sequences in the UniProtKB dataset. The Task 1 classifier identified 9,634 sequences as P-type ATPases. The classification in Task 2 (Figure 1) resulted in categorization of 9,477 of these 9,694 sequences into the 11 known classes (Table 3). The remaining 217 sequences (approximately 2.2%) were rejected by all 11 Task 2 classifiers and were placed in class 0. A list of these sequences is given in Table S9 in File S1. More information about the sequences is available online in our database (available via http://www.pumpkin.au.dk/pump-classifier/) that stores the results of the SLR-classifiers.

**Table 3:** Breakdown of P-type ATPase classes found in the UniProtKB dataset.

Domain/Kingdom	0	IA	IB	IIA	IIB	IIC	IID	IIIA	IIIB	IV	VA	VB	Total
Animal	49	1	68	165	102	248	2	0	0	251	35	63	984
Plant	9	2	156	66	134	4	0	179	1	108	14	2	675
Fungi	22	3	166	204	94	18	62	124	5	255	58	63	1074
One-celled Eukaryotes	48	1	57	77	81	22	12	32	1	199	33	79	642
–													–
Eukaryota	128	7	447	512	411	292	76	335	7	813	140	207	3375
Bacteria	79	489	3914	1042	38	72	29	38	362	27	0	1	6091
Archaea	8	7	133	35	0	14	0	29	0	0	0	0	226
Virus	2	0	0	0	0	0	0	0	0	0	0	0	2
													
*Total*	*217*	*503*	*4494*	*1589*	*449*	*378*	*105*	*402*	*369*	*840*	*140*	*208*	*9694*

Of the 9,694 sequences, 3,375 are eukaryotic, 6,091 bacterial, 226 archaeal and two come from virus. P-type ATPases are clearly highly represented in all domains of life. Somewhat surprising, two putative calcium P-type ATPases are found in a virus genome (*Paramecium bursaria chlorella*). These sequences (A7UR5 and A7CN8) could represent bacterial contaminations. However, they are quite identical (64%) but only 36% identical to ACA8 from *Arabidopsis*, which strongly suggests that they are not bacterial contaminations. Class IIC is not exclusive to animals in eukaryotes, but is widely represented in fungi, aveolata, protists and primitive plants (Table 3 and as reported recently by using other methods [33], [48]). Furthermore, class IIC ATPases were identified in prokaryotes where we observe 14 archaeal and 72 bacterial class IIC ATPases. Also, we observe seven eukaryotic sequences in class IA and also seven in class IIIB, two classes normally restricted to prokaryotes. Three of these sequences are fragments and might represent false positives, but most are worth further study. One bacterial sequence is classified as type VB but manual inspection indicate that the sequence rightfully belongs to IIA.

We also found 23 out of 840 sequences proposed to be class IV that in a phylogenetic tree of the 840 proposed class IV ATPases grouped in a cluster together with outgroups of other P-type ATPase subfamilies (Figure S5 in File S1). Several but not all of these sequences were bacterial. A manual inspection revealed that two bacterial sequences showed highest blast score to class IB ATPases, two eukaryotic sequences to class V ATPases, six sequences to secretory pathway Ca^2+^-ATPases (a subgroup of class 2A ATPases) and the remaining sequences grouped with other class 2A ATPases. In all cases, the scores for class IIA and class IV were similar as the sequences contain elements matching predictors from both these classes. In addition, these sequences cluster in the MSA-NJ in the subbranch belonging to class IIA (Figure S2 in File S1). We therefore speculate that they are specialized class IIA and other pumps with some elements that resemble class IV. *The power of the SLR method is highlighted here, since individual classifier-results following phylogenetic tree building can be rapidly compared and understood on the basis of the predictors*.

### SLR results compared to MSA-NJ

To further justify the SLR method and validate the SLR-classifiers' output on the UniProtKB dataset, the Task 2 classification of the 9,694 sequences was compared to a neighbour-joining (NJ) tree of the 9,694 sequences constructed from a multiple sequence alignment (MSA). The constructed NJ-tree shows a fairly nice distribution of the 11 classes (Figure S2 in File S1). The SLR classification and the NJ-tree grouping have excellent agreement, with 91.4% of the sequences classified by SLR clustered class-wise together within distinct subbranches of the NJ-tree. Excluding superclass II (i.e. class IIA and class IIB), the agreement increases to 96.7%, emphasizing that the overlap between SLR and MSA-NJ in general is very high, and that superclass II has some problems in the MSA-NJ analysis (discussed further below).

Despite a low number of positive training sequences for class IA and IIIB, the result appears robust. The IA classifier agrees almost perfectly with the MSA-NJ. The SLR-classifier finds 503 type IA and of these, 495 are located in one branch of the NJ tree. The agreement in class IIIB is less, but still quite good, with 343 of 369 hits located in the same sub-branch of the NJ-tree. Classes VA and VB are not clearly distinguishable in the NJ-tree, nor in the learning dataset (Figure S1 in File S1). These two classes probably should be merged to a single class when using full length sequences as the class differences are likely masked as opposed to the manual analysis of only core-fragments as in [14].

### Superclass II is ill-defined in the MSA-NJ analysis

Superclass II is not clearly divided in the MSA-NJ analysis. This is seen in Figure S2 in File S1, where the sub-tree grouping IIA, IIC and IID are not clearly separated. Using MSA-NJ, the only well defined class in superclass II appears to be the IIB class containing calmodulin-binding Ca^2+^ ATPases. Thus we cannot evaluate the SLR classification to a proper extent within this superclass. However, there is clearly a high disagreement between MSA-NJ and SLR for classes IIA (15.1% of sequences in the IIA group are classified as other classes by SLR), IIC (19.9%) and especially IID (56.3%), indicating that SLR at least disagrees with the faulty MSA-NJ classification. Class IID however also had a low number of positive training sequences (see Methods Section) and SLR might struggle if the initial training groups are not well-defined. For class IIB, the only clearly separated class in the MSA-NJ tree within superclass II, the disagreement between SLR and MSA-NJ is very low (0.7%) as it is for the other superclasses as stated in the previous section.

### Analysis of unclassified P-type ATPases

A small number of identified P-type ATPase sequences (217 of 9,694 (2.2%)) were rejected by all 11 SLR-classifiers. All cluster within subtrees with clear grouping in the NJ-tree (Figure S2 in File S1) indicating that these sequences could belong to that particular group. Thus we do not observe any new classes of P-type ATPases in this study. The fact that SLR only fails to classify 2.2% of all sequences in a highly divergent protein family demonstrates the power of the algorithm for this bioinformatics application. Why sequences could not be classified is not obvious. After building a NJ-tree of sequences in Class 0 and subsequent manual inspection (Figure S4 in File S1), we noticed that some subfamilies more often than others were misclassified. Thus, among the 217 unclassifiable P-type ATPase sequences, about 40 sequences grouped as class IIC ATPases (Na^+^/K^+^ pumps), 62 sequences grouped as class IIB ATPases (autoinhibited calmodulin-stimulated Ca^2+^-ATPases) and 46 grouped as class IIIA ATPases (putative Mg^2+^ pumps). Individual blast searches for all sequences confirmed this classification. Apparently, specific sequence features for these subfamilies need to be defined more stringently for the SLR algorithm to work optimal. Misclassification of sequences might be due to the use of small sequence motifs (1- 3 residues; Table S3 in File S1) as predictors. This is an inborn problem with analysis of highly variant protein families as conserved “motifs” often are single residues only placed in a conserved distance from other more easily identifiable sequences. (We note that SLR's predictors can be restricted by the user to be of or above a given length, an option potentially useful for some classification problems, but not employed here.)

### Variations on the canonical DKTGT motif

Since SLR does not rely on pre-defined motifs *per se* to identify potential P-type ATPases, it was possible to search the SLR-identified sequences for ATPases lacking the DKTGT defining motif [3]. Five eukaryotic and 17 bacterial sequences were found containing single point mutations in the DKTGT motif, which are clearly P-type ATPases based on BLAST search [44] (Table S3 in File S1). Some of these might derive from trivial sequencing-errors, or represent non-functional or highly specialized P-type ATPases, 99.77% of identified P-type ATPases contain the DKTGT motif which supports the known result that P-type ATPases are strongly characterized by this motif [1].

### Analysis of the Genome dataset

Sequence fragments as well as lack of full genomic data for most species in the UniProtKB dataset complicate the analysis of organismal P-type ATPase distribution. For this, we turned to the Genome dataset containing 70 eukaryotic, 975 bacterial and 78 archaeal genomes downloaded from Entrez. Here 5,821 ATPases were identified. Table S5 in File S1 shows the overall class-distribution that is similar to the distribution in the UniProtKB dataset. The Genome dataset was downloaded at a later time than the UniProtKB dataset, and it thus contains more sequences in a few classes. Examples of the organismal distribution of some selected species can be seen in Table S6 in File S1.

#### Eukaryotic P-type ATPases

**Figure 2:**
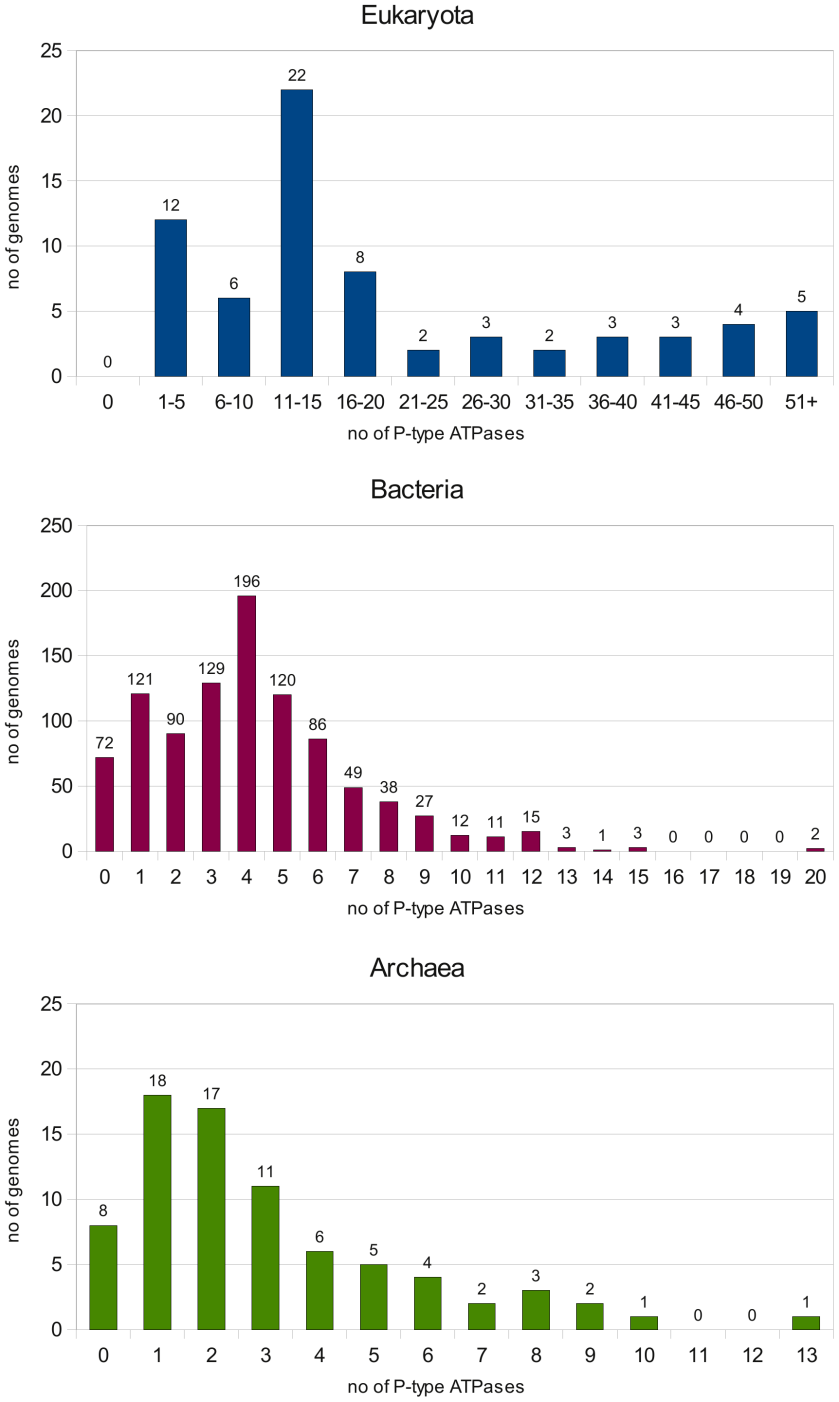
Distribution of the total number of P-type ATPases inc. isoforms found in individual genomes in the Genome dataset. Eukaryota (n = 70), Bacteria (n = 975) and Archaea (n = 78).

Eukaryotic organisms have considerably more P-type ATPases than bacteria and archaea (Figure 2), and numbers are higher than reported previously (PAT-base; [12]), caused by the inclusion of isoforms, giving a detailed view of the diversity and density of P-type ATPases in eukaryotes.

A problem with automated searching tools like HMM, SVM or SLR is that their performance depends on the level of redundancy in the databases. Especially highly similar eukaryotic sequences from the same locus may have been entered on several occasions. Manual inspection is often required to distinguish true gene duplications from true gene redundancy. An extreme example is *Canis lupus familiaris* that as a result of an SLR analysis of the dataset has 166 P-type ATPases (Table S6 in File S1), a number which following manual inspection of the sequences could be reduced to 37 (compared to 36 in humans) (Table S7 in File S1).

Even though application of the SLR method without subsequent sorting for redundancy overestimates the true number of pumps, a large variety of P-type ATPases, especially of type IV, are clearly important for multicellular organisms (plants and animals) (Table S5 in File S1). Furthermore, plants have a high number of class IIIA H^+^ P-type ATPases and animals a high number of class IIC Na^+^/K^+^ ATPases as expected as these pumps have an analogous function in energizing the plasma membrane. Interestingly, some fungi contain both Na^+^/K^+^ ATPases and plasma membrane H^+^-ATPases.

#### Bacterial P-type ATPases

Bacterial taxonomy is quite diverse and overall the number of P-type ATPases ranges from 1 to 12 per genome (Figure 2). Most are class IA, IB, IIA and IIIB (Table S6 in File S1). The large number of IIIB sequences in bacteria (Table 3 and Table S6 in File S1) is remarkable and suggests a more central role of this class than previously appreciated. Also remarkable 52 class IIC ATPases are found. 11 class IV ATPases are observed, but like for the UniProtKB dataset they are found to be false positives upon manual inspection, rather belonging to class IIA. Similarly, a single VA ATPase is found which seems to belong to class IIA.

#### Archaeal P-type ATPases

Most archaea have a low number of P-type ATPases, in the range of 1 to 3 (Figure 2), and these are mostly class IB as well as some class IIIA (Table S6 in File S1). 17 class IIC ATPases are found here.

### Genomes without P-type ATPases

The Genome dataset consists of 1,123 genomes of which 1,043 (92.9%) contain P-type ATPases, including all eukaryotic species. 80 genomes representing 60 different species are lacking P-type ATPases altogether (8 archaeal and 72 bacterial, Table S8 in File S1). Some genomes are the only ones sequenced within their genus, and it therefore remains to been seen if the lack of P-type ATPases is a general feature of those particular evolutionary branches.

Some genera clearly survive without P-type ATPases. These primarily include the order *Rickettsiales* with the following genera: *Anaplasma*, *Ehrlichia*, *Neorickettsia*, *Orientia*, *Rickettsia* and *Wolbachia* (29 genomes). Also the genera *Bartonella*, *Borrelia*, *Buchnera* and *Xylella* do not have P-type ATPases (23 genomes). All of the above-mentioned genera are obligate endosymbionts, which may explain why they can survive without these vital pumps. A number of genera contain a mix of genomes with and without P-type ATPases. These include *Campylobacter*, *Coxiella*, *Francisella*, *Haemophilus*, *Helicobacter* and *Mycoplasma* as well as several uncharacterized species (*Candidatus*)

### Membrane topology analysis

**Figure 3:**
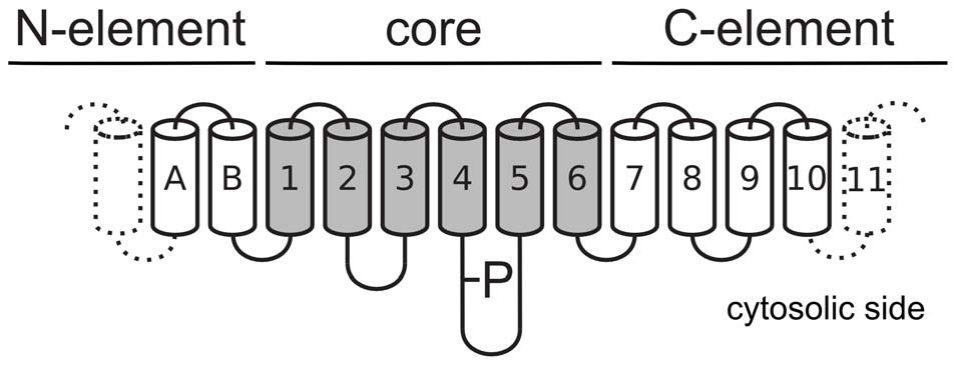
Membrane Topology in P-type ATPases. **Top:** Overview of the membrane topology found in P-type ATPases. Gray helices denote the 6 TM core-element found in all pumps (here numbered 1–6). P shows the cytosolic phosphorylation site containing the DKTGT motif.

We analyzed the transmembrane topology of the P-type ATPases found in both the UniProtKB and Genome datasets (full data available online). A pattern emerged which became much clearer if simplified to three transmembrane elements instead of the exact number of transmembrane helices (Figure 3). The three elements in P-type ATPases are: A core-element of 6 transmembrane helices [10], [45], and an N- and C-terminal element. The observed topology in the Genome dataset is summarized in Table 4. On the C-terminal side of the core an extension of 4 transmembrane helices is often found (e.g., Ca^2+^-ATPase, Na^+^, K^+^-ATPase, H^+^-ATPase). This element can be expanded further (e.g, SERCA2B has 11 transmembrane helices [34]), and some sequences are predicted to have up to 17 transmembrane helices in the C-terminal element for at total of 23 transmembrane helices. These long sequences are ATPases fused C-terminally to mononucleotidyl cyclases [35]. On the N-terminal side an extension of 2 transmembrane helices is often found. Like the C-element, this element can have additional number of helices reaching a total of 22 transmembrane helices. These longer ones appear to be ATPases fused N-terminally to Kef-type K^+^ transport systems [36]. Some ATPases (particularly superclass V) have both the N- and C-terminal element, whereas most have only one. As a generalization, class IA has a 7 TM topology, class IB contains the N-terminal element, class VA and VB have the N- and C-terminal elements, and all other classes contain only the C-terminal element. A subclass of IB ATPases (predicted Co-ATPases [37]) are reduced to the 6 TM core.

**Table 4:** Membrane Topology in P-type ATPases.

Class	core only	N-core	core-C	N-core-C	core-1TM	Broken core	Total
0	3	1	40	7	-	16	67
IA	63	3	4	22	312	10	414
IB	128	2121	105	12	-	476	2842
IIA	3	0	879	8	-	46	936
IIB	5	0	302	10	-	14	331
IIC	3	0	157	1	-	10	171
IID	0	0	49	1	-	4	54
IIIA	0	0	123	3	0	29	155
IIIB	3	3	230	7	-	22	265
IV	1	0	357	21	-	57	436
VA	0	0	10	35	-	6	51
VB	0	0	18	69	-	12	99
*Total*	*209*	*2128*	*2274*	*196*	*312*	*702*	*5821*

‘N’ and ‘C’ denote the N- and C-terminal element respectively. ‘Core-1TM’ denotes proteins with exactly one TM after the core (only class IA). ‘Broken core’ counts sequences with less than 6 TM in the core, regardless of total number of TMs.

Pump functionality is contained within the core-element, while the N- and C-terminal elements function as stabilizing elements, being heavily involved in regulation and interaction with other subunits [45]. A few sequences with missing transmembrane helices in the core-element are presumably broken gene products. The observation that some ATPases, especially in superclass V, have both the C- and N-terminal element emphasizes that these extensions do not occupy the same position in the membrane space (Figure S3 in File S1). This is consistent with the short linkers observed from core to the N-terminal extension in IB ATPases [38], [39].

## Conclusions

In this paper we applied a new machine learning tool, Structured Logistic Regression, to the problem of large scale identification and categorization of P-type ATPases. We show that SLR is efficient and useful for this task.

A number of factors speak to SLR's advantage compared to HMM for the initial identification of P-type ATPases (Task 1): Profile HMM's need sequence alignment for time efficient training. SLR requires no alignments, and focuses directly on separating the given classes, making SLR independent of other methods. Furthermore the testing time on UniProtKB Dataset for HMM is 150 minutes compared to 19 minutes for SLR. Given the fast-paced increase of the UniProtKB this may become a considerable gap in the future. Finally HMM seem to lead to a surprisingly high number of false positives when filtering out a particular type of proteins. Arguably this can be improved by redefining the HMM thresholds, but it is preferable to not manually optimise parameters when doing large scale analysis (e.g. Pfam [43]). SLR is more conservative, and thus may lose some sequences, but the retrieved set has very few false positives, even without manual optimization. Minimising the FP-rate is significant, since even a small fraction of false positives will lead to a large influence on the final classification result as positive hits are normally on a much smaller scale that the negatives for a given class in bioinformatics (needle-in-a-haystack problem). Presumably the use of both positive and negative datasets in the training of SLR gives it an advantage over HMM that are trained using only positive sequences. We have chosen not to compare SLR to SVM, since this comparison has already been performed in [23] for string classification using a smaller dataset (thousands, not millions of test-cases). This comparison showed that SLR and SVM are equally accurate, but SLR is order of magnitudes more scalable. Additionally SVM typically restricts the maximum size of k-mers (i.e., the predictors are restricted to sub-sequences up to length k, for small k = 3 or k = 10) due to these computational considerations, while SLR works with arbitrary long predictors.

The MSA-NJ analysis of the P-type ATPases identified in Task 1 shows that the classes observed 12 years ago are surprisingly robust when exposed to the large number of new P-type ATPase sequences found. The agreement between the MSA-NJ analysis and SLR classification into subclasses in Task 2 validates the SLR based classification. It is important to emphasize that we do not suggest MSA-NJ analysis as an alternative to SLR for classification. We use MSA-NJ only to validate the classification performed by SLR when the number of sequences to classify are sufficiently small for this to be computational feasible. Constructing NJ-trees is an established technique in the bioinformatics community for hierarchical clustering of a set of items (fx represented by sequences) where pairwise distances are known (or can be inferred). Given the pairwise distances, a NJ-tree of set items can be computed in time cubic in the number of items. Using a faster implementation of the canonical method, fx RapidNJ [47], or heuristics that do not guarantee to construct a true NJ-tree, fx ClearCut [46], can speed up the computation such that it in practice becomes (close to) quadratic in the number of items. However, before constructing the NJ-tree, we must infer the pairwise distances between the items. For sequence data, where each item corresponds to a sequence, this often involves constructing a multiple sequence alignment from which the pairwise distances are inferred. By using heuristics, fx MUSCLE [27] or MAFTT [29], this is reasonable fast in practice but still significantly slower than the subsequent construction of a NJ-tree. The process of constructing a multiple sequence alignment and a NJ-tree can be compared to training the SLR and it takes comparable time. However, training a SLR, results in a high scalable classifier that can be used for classification of new sequences (or sequence fragments) based on the knowledge obtained during training.

The SLR-based classification of P-type ATPases provides a footing for further biochemical analysis of this interesting protein-family. We have provided the methodology and online tool to categorize new sequences as well as identified more than 10,000 sequences as P-type ATPases all available online for further data-mining. Several protein sequences emerged during this analysis that seem promising as targets for further functional characterization and crystallization trials. Non-metazoan homologues of Na^+^/K^+^ and H^+^/K^+^ ATPases are obvious examples. Identifying fungal and prokaryotic members of this essential class could aid understanding of functionality by allowing for cloning, over-expression and mutational research strategies with greater ease than associated with the mammalian orthologues. Also fungal organisms containing both IIC and IIIA ATPases were found (e.g. *Aspergillus fumigatus*), which is unexpected as these two classes usually have an analogous function in the cell membrane in maintaining the transmembrane potential [33], [45].

Class IIIB of Mg-importers is much larger than previously reported, highlighting its important, but largely uncharacterized, contribution to magnesium homeostasis in bacteria. Further biochemical characterization of this group seems necessary. While SLR proposed a small number of class IV and V pumps in bacteria, we analysed these manually and derive that they are false positives, caused by poor division of superclass II. We conclude that no P-type ATPases of the class IV and V are found in bacteria, and these classes thus represent more recent evolutionary achievements. The lack of type IV P-type ATPases (putative lipid flippases proposed to be involved in formation of secretory vesicles) in prokaryotes most likely reflects the fact that these cells lack internal membrane systems and a secretory pathway to maintain them.

Only a small number of sequences could not be assigned to the existing 11 classes, and no new class of P-type ATPases is observed in our investigation. This work also highlights that superclass II might benefit from a detailed analysis to better identify and separate classes. Our database provides the foundation to perform such future dissection.

The analysis of membrane topology based solely on sequences is difficult [40]. However, with the problem simplified to TM-elements, a clear pattern emerges for P-type ATPases, highlighting the importance of the central 6 transmembrane helices found in all pumps. Analysing the topology of more than 10,000 P-type ATPases, we note that all contain a core-element of 6 transmembrane helices flanked by optional N- and C-terminal elements that contribute to stability, regulation and that may confer new functionalities to the protein. The opening and release of the exported cations require exactly M1-2, M3-4 and M5-6 of the core to separate as observed in the Ca^2+^-ATPase [8] highlighting that the actual transport function is retained within the core [45]. Furthermore, the cation binding site is defined mainly by M4, M5, and M6 in all structures solved, and this also appears to be the case in heavy metal ATPases [4], [6], [37], [41], [42].

Finally, we emphasize that SLR can be used on an even larger scale than in the P-type ATPase application shown here. For instance, one could envision using SLR to classify large databases according to protein families as implemented in Pfam using HMM [43]. The availability of the SLR predictors, as well as scores for the individual SLR classifiers means that it is easy to comprehend, compare and evaluate a proposed prediction. We encourage the use of SLR in large-scale analysis of other protein-families by the methodology presented here.

## Supporting Information

File S1 **Supplementary Tables and Figures.**
(DOC)
