# Supplementary material for: Large Scale Identification and Categorization of Protein Sequences Using Structured Logistic Regression
Source: PLoS One. 2014 Jan 20;9(1):e85139. doi: 10.1371/journal.pone.0085139 (PMC3896382; doi:10.1371/journal.pone.0085139)
Supplement: File S1 — Supplementary Tables and Figures. (DOC) [file pone.0085139.s001.doc]

# Large scale identification and categorization of proteins sequences using Structured Logistic Regression

Bjørn P. Pedersen^1,2#*^, Georgiana Ifrim^7#^, Poul Liboriussen^1,3#^, Kristian B. Axelsen^1,4^, Michael G. Palmgren^1,5^, Poul Nissen^1,2^, Carsten Wiuf ^6^, Christian N. S. Pedersen^1,3*^

^1^Centre for Membrane Pumps in Cells and Disease - PUMPKIN, Danish National Research Foundation.

^2^Department of Molecular Biology, Aarhus University, 8000 Aarhus C, Denmark.

^3^Bioinformatics Research Centre, Aarhus University, 8000 Aarhus C, Denmark.

^4^Swiss-Prot Group, Swiss Institute of Bioinformatics, CMU, 1 rue Michel-Servet, CH-1211 Geneva 4, Switzerland.

^5^Department of Plant and Environmental Sciences, University of Copenhagen, 1871 Frederiksberg C, Denmark.

^6^Department of Mathematical Sciences, University of Copenhagen, 2100 Copenhagen Ø, Denmark.

^7^INSIGHT Centre for Data Analytics, University College Dublin, Ireland.

#These authors contributed equally to this work.

*Corresponding authors.

Email addresses:

BPP: bpp@mb.au.dk

GI: georgiana.ifrim@ucd.ie

PL: poul.liboriussen@gmail.com

KBA: kax@life.ku.dk

MGP: palmgren@life.ku.dk

PN: pn@mb.au.dk

CW: wiuf@math.ku.dk

CNSP: cstorm@birc.au.dk

**Supplementary Table 1.**Average AUC scores for SLR over 1,000 random data splits with 90% training and 10% test for Task 2.

| Classifier | IA | IB | IIA | IIB | IIC | IID | IIIA | IIIB | IV | VA | VB |
| --- | --- | --- | --- | --- | --- | --- | --- | --- | --- | --- | --- |
| SLR Average AUC | 95.28% | 99.58% | 99.96% | 99.92% | 98.47% | 93.49% | 98.67% | 89.32% | 99.96% | 99.95% | 99.74% |

**Supplementary Table 2.**Average TP and FP scores for SLR over 1,000 random data splits with 90% training and 10% test for Task 2.

| SLR-classifier | no. of sequences in dataset (True pos. / True neg.) | % TP | % FP | % FN | % TN |
| --- | --- | --- | --- | --- | --- |
| IA | 12 / 478 | 90.60 | 0.09 | 9.40 | 99.91 |
| IB | 112 / 378 | 96.38 | 0.39 | 3.62 | 99.61 |
| IIA | 56 / 434 | 96.80 | 0.13 | 3.20 | 99.87 |
| IIB | 32 / 458 | 99.92 | 0.00 | 0.07 | 100.00 |
| IIC | 47 / 443 | 97.14 | 0.10 | 2.86 | 99.90 |
| IID | 8 / 482 | 89.30 | 0.26 | 10.70 | 99.74 |
| IIIA | 59 / 431 | 93.52 | 0.34 | 6.48 | 99.66 |
| IIIB | 4 / 486 | 82.10 | 0.92 | 17.90 | 99.08 |
| IV | 50 / 440 | 96.64 | 0.33 | 3.36 | 99.67 |
| VA | 40/ 450 | 97.65 | 0.05 | 2.35 | 99.95 |
| VB | 70 / 420 | 95.33 | 0.24 | 4.67 | 99.76 |

**Supplementary Table 3.**Predictors and weights used by the SLR-classifiers.

| SLR-classifier | No. of sequences in training set (True pos. / True neg.) | Weight & Predictor | SLR-classifier | No. of sequences in training set (True pos. / True neg.) | Weight & Predictor |
| --- | --- | --- | --- | --- | --- |
| P-type | 490 / 43,315 | 0.411 DKTGT  0.121 TGTL  0.109 VGD  0.084 VFI  0.078 GDG  0.069 KGA  0.061 LVI  0.059 TGD  0.058 TGE  -0.052 GP  -0.067 PH  -0.101 S  -0.263 M | IIIA | 59/431 | 0.176 LCSD  0.101 GMTG  0.093 VPGD  0.079 GIAV  0.076 VFP  0.066 FDP  0.064 KRT  0.060 PFDP  0.055 PPR  0.052 VDK  -0.072 AMG  -0.094 KTG |
| IA | 12 / 478 | 0.717 TPNE  0.253 PTTI  0.114 RSI  0.073 GDN  -0.094 LGL  -0.134 TGTL  -0.254 LE  -0.258 A | IIIB | 4/486 | 1.334 PEMLP  0.272 EMLP  -0.054 QN  -0.061 GG  -0.071 TL  -0.158 A |
| IB | 112 / 378 | 0.089 PCA  -0.084 EMLP | IV | 50/440 | 0.079 SPDE  0.065 LVHG  -0.875 GIYD |
| IIA | 56 / 434 | 0.040 MTGDGVN  0.032 LAVAA  -0.065 LVHG  -0.092 VTGDG  -0.716 GIYD | VA | 40/450 | 1.241 CGDGTNDVGA  0.296 CTEP  0.050 SQG  -0.085 LG  -0.138 A |
| IIB | 32 / 458 | 1.310 CETM  -0.689 A |  |  |  |
| IIC | 47 / 443 | 0.530 VTGDGV  0.340 FFST  0.075 VTGDG  0.058 TVT  0.052 VTGD  -0.051 AC  -0.058 DKT  -0.070 VAA  -0.074 SPT  -0.083 TGT  -0.142 A  -0.178 GDG | VB | 70/420 | 0.178 PPALP  0.141 NDC  0.088 CGDG  0.081 CFDKT  0.079 FDKTGT  0.057 LC  -0.054 CP  -0.064 EGL  -0.065 NDA  -0.093 FAG  -0.094 TEP  -0.121 P |
| IID | 8/482 | 1.227 GIYD  -0.772 A |  |  |  |

Predictions with weight <|0.05| have been excluded from this list for brevity. A complete list can be seen online.

**Supplementary Table 4.**Variations on the DKTGT motif found in the UniProtKB dataset.

| Motif | # of sequences | Taxonomy | Species | Predicted class | UniProtKB accession number |
| --- | --- | --- | --- | --- | --- |
| DKTGT | 9671 | - | - | - | - |
| D**LV**GT | 1 | Eukaryota :: Metazoa | *Drosophila melanogaster* | IIC | q9w248 |
| D**E**TGT | 1 | Eukaryota :: Fungi | *Pichia angusta* | IIIA | o94195 |
| DKTG**N** | 2 | Eukaryota :: Fungi | 2 *Ajellomyces dermatitidis* | IIA | c5ght8; c5jf70 |
| DKTG**A** | 1 | Eukaryota :: Aveolata | *Oxytricha trifallax* | 0 | q6rxx1 |
| DKTG**V** | 5 | Bacteria :: Proteobacteria | 2 *Campylobacter sp.* | IB | q4hgu4; b9kfi5 |
|  |  |  | 2 *Caulobacter sp.* | IB | b0svv2; b8h586 |
|  |  |  | *Phenylobacterium zucineum* | IB | b4r899 |
| DKTG**I** | 12 | Bacteria :: Proteobacteria | 11 *Campylobacter jejuni ssp.* | IB | q7bpr8; q6tg04; q0p9a1; q5htv6; a7h2l2; a3ypa8; a3zhk4; a3ymn7; b5qg19; a5kfy6; a8fml3 |
|  |  |  | *Campylobacter upsaliensis* | IB | q4htp2 |

**Supplementary Table 5.**Number of different P-type ATPases found in different kingdoms and Phylum using the genome dataset.

| Kingdom/Phylum | 0 | IA | IB | IIA | IIB | IIC | IID | IIIA | IIIB | IV | VA | VB | Total | # of genomes analyzed with hits * |
| --- | --- | --- | --- | --- | --- | --- | --- | --- | --- | --- | --- | --- | --- | --- |
| Animal | 8 | 0 | 45 | 143 | 200 | 96 | 0 | 0 | 0 | 260 | 15 | 69 | 836 | 24 |
| Plant | 3 | 0 | 63 | 21 | 72 | 1 | 0 | 56 | 1 | 47 | 7 | 0 | 271 | 7 |
| Fungi | 10 | 1 | 39 | 28 | 27 | 2 | 25 | 28 | 3 | 74 | 17 | 17 | 271 | 24 |
| One-celled Eukaryota | 6 | 2 | 21 | 27 | 21 | 3 | 6 | 12 | 3 | 44 | 11 | 13 | 169 | 15 |
| **Total Eukaryota** | **27** | **5** | **185** | **219** | **320** | **102** | **31** | **96** | **7** | **425** | **50** | **99** | **1547** | **70** |
|  |  |  |  |  |  |  |  |  |  |  |  |  |  |  |
| Proteobacteria | 7 | 245 | 1213 | 182 | 0 | 16 | 5 | 18 | 176 | 3 | 0 | 0 | 1865 | 436 |
| Cyanobacteria | 4 | 14 | 92 | 41 | 1 | 5 | 0 | 1 | 1 | 0 | 0 | 0 | 159 | 38 |
| Spirochetes | 0 | 2 | 22 | 2 | 0 | 2 | 1 | 0 | 1 | 0 | 0 | 0 | 30 | 10 |
| Chlamydias | 0 | 3 | 26 | 0 | 1 | 0 | 0 | 1 | 0 | 0 | 0 | 0 | 32 | 18 |
| Firmicutes | 11 | 73 | 555 | 333 | 1 | 9 | 10 | 0 | 56 | 4 | 0 | 0 | 1052 | 198 |
| Other bacteria | 12 | 67 | 625 | 124 | 8 | 20 | 6 | 8 | 24 | 4 | 1 | 0 | 899 | 203 |
| **Total Bacteria** | **34** | **404** | **2534** | **682** | **11** | **52** | **22** | **28** | **258** | **11** | **1** | **0** | **4037** | **903** |
|  |  |  |  |  |  |  |  |  |  |  |  |  |  |  |
| Crenarchaea | 2 | 0 | 23 | 2 | 0 | 0 | 0 | 10 | 0 | 0 | 0 | 0 | 37 | 22 |
| Euarchaeota | 4 | 7 | 116 | 33 | 0 | 17 | 1 | 20 | 0 | 0 | 0 | 0 | 198 | 47 |
| Korarchaeota | 0 | 0 | 1 | 0 | 0 | 0 | 0 | 1 | 0 | 0 | 0 | 0 | 2 | 1 |
| **Total Archaea** | **6** | **7** | **140** | **35** | **0** | **17** | **1** | **31** | **0** | **0** | **0** | **0** | **237** | **70** |
|  |  |  |  |  |  |  |  |  |  |  |  |  |  |  |
| **Total** | **67** | **416** | **2859** | **936** | **331** | **171** | **54** | **155** | **265** | **436** | **51** | **99** | **5821** | **1043** |

* A total of 1123 genomes was analyzed. 80 genomes did not contain P-type ATPases, and are listed in Supplementary Table 6.

**Supplementary Table 6.**Examples of organismal P-type ATPase distribution from selected kingdoms and phylum.

| Kingdom/Phylum | Species | 0 | IA | IB | IIA | IIB | IIC | IID | IIIA | IIIB | IV | VA | VB | Total |
| --- | --- | --- | --- | --- | --- | --- | --- | --- | --- | --- | --- | --- | --- | --- |
| Animal | *Anopheles gambiae* | 0 | 0 | 1 | 6 | 1 | 1 | 0 | 0 | 0 | 5 | 1 | 1 | 16 |
|  | *Caenorhabditis elegans* | 0 | 0 | 1 | 6 | 7 | 5 | 0 | 0 | 0 | 11 | 1 | 6 | 37 |
|  | *Canis Lupus familiaris* | 1 | 0 | 4 | 26 | 103 | 7 | 0 | 0 | 0 | 20 | 1 | 4 | 166 |
|  | *Drosophila melanogaster* | 0 | 0 | 2 | 13 | 6 | 10 | 0 | 0 | 0 | 21 | 1 | 4 | 57 |
|  | *Homo sapiens* | 0 | 0 | 7 | 17 | 6 | 7 | 0 | 0 | 0 | 17 | 1 | 6 | 61 |
|  | *Pan troglodytes* | 1 | 0 | 0 | 19 | 14 | 5 | 0 | 0 | 0 | 35 | 0 | 7 | 81 |
| Plant | *Arabidopsis thaliana* | 0 | 0 | 10 | 4 | 10 | 0 | 0 | 12 | 0 | 12 | 1 | 0 | 49 |
|  | *Oryza sativa* | 1 | 0 | 9 | 2 | 10 | 0 | 0 | 10 | 0 | 4 | 1 | 0 | 37 |
|  | *Populus trichocarpa* | 0 | 0 | 11 | 4 | 12 | 0 | 0 | 10 | 0 | 8 | 1 | 0 | 46 |
|  | *Vitis vinifera* | 0 | 0 | 7 | 3 | 25 | 0 | 0 | 10 | 0 | 11 | 0 | 0 | 56 |
| Fungi | *Penicillium chrysogenum* | 2 | 0 | 8 | 3 | 5 | 0 | 2 | 4 | 0 | 4 | 1 | 1 | 30 |
|  | *Saccharomyces cerevisiae* | 0 | 0 | 2 | 1 | 1 | 0 | 3 | 2 | 0 | 5 | 1 | 1 | 16 |
|  | *Schizosaccharomyces pombe* | 0 | 0 | 0 | 1 | 1 | 0 | 1 | 2 | 0 | 5 | 1 | 2 | 13 |
| one-celled | *Leishmania infantum* | 0 | 0 | 1 | 1 | 4 | 0 | 1 | 2 | 0 | 5 | 1 | 0 | 15 |
|  | *Plasmudium falciparum* | 1 | 0 | 1 | 2 | 0 | 0 | 1 | 0 | 0 | 3 | 0 | 2 | 10 |
|  | *Trypanosoma brucei* | 0 | 0 | 1 | 1 | 4 | 0 | 1 | 2 | 0 | 5 | 0 | 0 | 14 |
|  |  |  |  |  |  |  |  |  |  |  |  |  |  |  |
| Actinobacteria | *Mycobacterium marinum* | 0 | 1 | 16 | 2 | 0 | 0 | 0 | 1 | 0 | 0 | 0 | 0 | 20 |
| Cyanobacteria | *Anabaena variabilis* | 0 | 2 | 7 | 4 | 0 | 1 | 0 | 0 | 1 | 0 | 0 | 0 | 15 |
| Chlamydias | *Chlamydia trachomatis* | 0 | 0 | 1 | 0 | 0 | 0 | 0 | 0 | 0 | 0 | 0 | 0 | 1 |
| Firmicutes | *Enterococcus faecalis* | 0 | 1 | 6 | 4 | 0 | 0 | 0 | 0 | 2 | 0 | 0 | 0 | 13 |
|  | *Staphylococcus aureus* | 0 | 1 | 1 | 0 | 0 | 0 | 0 | 0 | 0 | 0 | 0 | 0 | 2 |
|  | *Streptococcus pneumoniae* | 0 | 0 | 2 | 2 | 0 | 0 | 0 | 0 | 0 | 0 | 0 | 0 | 4 |
|  | *Clostridium botulinum* | 0 | 0 | 3 | 4 | 0 | 0 | 0 | 0 | 1 | 0 | 0 | 0 | 8 |
| Mycoplasmas | *Mycoplasma genitalium* | 0 | 0 | 0 | 0 | 0 | 1 | 0 | 0 | 0 | 0 | 0 | 0 | 1 |
| Proteobacteria | *Agrobacterium radiobacter* | 0 | 1 | 5 | 0 | 0 | 0 | 0 | 0 | 0 | 0 | 0 | 0 | 6 |
|  | *Neisseria meningitidis* | 0 | 0 | 2 | 0 | 0 | 0 | 0 | 0 | 0 | 0 | 0 | 0 | 2 |
|  | *Desulfovibrio vulgaris* | 0 | 1 | 3 | 1 | 0 | 0 | 0 | 0 | 0 | 0 | 0 | 0 | 5 |
|  | *Salmonella enterica* | 0 | 1 | 2 | 0 | 0 | 0 | 0 | 0 | 2 | 0 | 0 | 0 | 5 |
|  | *Escherichia coli* | 0 | 1 | 2 | 0 | 0 | 0 | 0 | 0 | 1 | 0 | 0 | 0 | 4 |
| Spirochetes | *Brachyspira hyodysenteria* | 0 | 0 | 2 | 0 | 0 | 0 | 1 | 0 | 1 | 0 | 0 | 0 | 4 |
| Thermotogae | *Thermotoga maritima* | 0 | 0 | 1 | 0 | 0 | 0 | 0 | 0 | 0 | 0 | 0 | 0 | 1 |
|  |  |  |  |  |  |  |  |  |  |  |  |  |  |  |
| Euarchaeota | *Archaeoglobus fulgidus* | 0 | 0 | 2 | 0 | 0 | 0 | 0 | 0 | 0 | 0 | 0 | 0 | 2 |
|  | *Halorubrum lacusprofundi* | 0 | 0 | 9 | 0 | 0 | 0 | 0 | 0 | 0 | 0 | 0 | 0 | 9 |
|  | *Methanocella paludicola* | 0 | 1 | 6 | 1 | 0 | 1 | 1 | 3 | 0 | 0 | 0 | 0 | 13 |
|  | *Methanococcus maripaludis* | 0 | 0 | 1 | 1 | 0 | 1 | 0 | 0 | 0 | 0 | 0 | 0 | 3 |
|  | *Thermoplasma acidophilum* | 0 | 1 | 1 | 0 | 0 | 0 | 0 | 1 | 0 | 0 | 0 | 0 | 3 |
| Crenarchaeota | *Staphylothermus marinus* | 0 | 0 | 2 | 0 | 0 | 0 | 0 | 0 | 0 | 0 | 0 | 0 | 2 |
|  | *Sulfolobus solfataricus* | 0 | 0 | 2 | 0 | 0 | 0 | 0 | 0 | 0 | 0 | 0 | 0 | 2 |
| Korarchaeota | *Candidatus Korarchaeum cryptofilum* | 0 | 0 | 1 | 0 | 0 | 0 | 0 | 1 | 0 | 0 | 0 | 0 | 2 |

The complete organismal P-type ATPase distribution from 1,123 genomes can be found online.

**Supplementary Table 7.** An in-depth analysis of the P-type ATPases in dog (*Canis Lupus familiaris*).

| **Gene loci** | **Class** | **Sequences** |
| --- | --- | --- |
| **ATP7A** | 1B | XP_549096, XP_860306 ^a^ |
| **ATP7B** | 1B | NP_001020438 |
| **ATP2A1** | 2A | XP_860120, XP_860010, XP_859765 ^a^, XP_860087 ^a^, XP_859936 ^a^, XP_536925 ^a^, XP_859832 ^a^, XP_859867 ^a^, XP_859903 ^a^, XP_859973 ^a^, XP_849777 ^a^ |
| **ATP2A2** | 2A | NP_001003214 |
| **ATP2A3** | 2A | XP_548558, XP_868561 ^a^, XP_854556 ^a^ |
| **ATP2C1** | 2A | XP_534262, XP_851493, XP_863744, XP_863766, XP_863813 ^a^, XP_863833 ^a^, XP_863679 ^a^, XP_863719 ^a^, XP_863698 ^a^, XP_863656 ^a^ |
| **ATP2C2** | 2A | XP_536762 |
| **ATP2B1** | 2B | XP_865786 ^b^, XP_865502 ^b^, XP_865769 ^b^, XP_865736 ^b^, XP_865591 ^b^, XP_865708 ^b^, XP_865662 ^b^, XP_865615 ^b^, XP_865723 ^b^, XP_852421 ^b^, XP_532647 ^b^, XP_865691 ^b^, XP_865643 ^b^, XP_865414 ^b^, XP_865384 ^b^, XP_865753 ^b^, XP_865600 ^b^, XP_865573 ^b^, XP_865557 ^b^, XP_865467 ^b^, XP_865450 ^b^, XP_865432 ^b^, XP_865397 ^b^, XP_865538 ^b^ |
| **ATP2B2** | 2B | XP_533742, XP_861223, XP_861393, XP_860934 ^a^, XP_860904 ^a^, XP_861021 ^a^, XP_860996 ^a^, XP_860379 ^a^, XP_860582 ^a^, XP_860345 ^a^, XP_860414 ^a^, XP_860711 ^a^, XP_860449 ^a^, XP_860545 ^a^, XP_860316 ^a^, XP_860783 ^a^, XP_860517 ^a^, XP_860643 ^a^, XP_860616 ^a^, XP_860845 ^a^, XP_860744 ^a^, XP_860810 ^a^, XP_860677 ^a^, XP_861334 ^a^, XP_861134 ^a^, XP_860276 ^a^, XP_860967 ^a^, XP_861163 ^a^, XP_861050 ^a^, XP_861104 ^a^, XP_861077 ^a^, XP_861279 ^a^, XP_861192 ^a^, XP_861249 ^a^, XP_860485 ^a^, XP_850399 ^a^, XP_860871 ^a^ |
| **ATP2B3** | 2B | XP_867106, XP_867228, XP_867258, XP_867143 ^a^, XP_549358 ^a^, XP_867152 ^a^, XP_867085 ^a^, XP_867269 ^a^, XP_867116 ^a^, XP_867030 ^a^, XP_866942 ^a^, XP_866932 ^a^, XP_867004 ^a^, XP_867040 ^a^, XP_866920 ^a^, XP_867010 ^a^, XP_866985 ^a^, XP_866995 ^a^, XP_867094 ^a^, XP_867209 ^a^, XP_867250 ^a^, XP_867188 ^a^, XP_867020 ^a^, XP_867060 ^a^, XP_867073 ^a^, XP_866972 ^a^, XP_866964 ^a^, XP_866954 ^a^, XP_867221, XP_867280 ^a^, XP_867198 ^a^, XP_867178 ^a^, XP_867168 ^a^, XP_867161 ^a^, XP_867239 ^a^, XP_853260 ^a^, XP_867050 ^a^ |
| **ATP2B4** | 2B | XP_855748, XP_855913, XP_855833 ^a^, XP_536090 ^a^, XP_848332 ^a^ |
| **ATP4A** | 2C | NP_001003342 |
| **ATP12A** | 2C | XP_848278 |
| **ATP1A1** | 2C | NP_001003306 |
| **ATP1A2** | 2C | XP_545753, XP_860689 ^a^ |
| **ATP1A3** | 2C | XP_855286 |
| **ATP1A4** | 2C | XP_545754 |
| **ATP8A1** | 4 | XP_849357, XP_858569 ^a^ |
| **ATP8A2** | 4 | XP_543162 |
| **ATP8B1** | 4 | XP_533394 |
| **ATP8B2** | 4 | XP_547569 |
| **ATP8B3** | 4 | XP_855076 |
| **ATP8B4** | 4 | XP_544674 |
| **ATP8B5** | 4 | XP_854716 |
| **ATP9A** | 4 | XP_534457 |
| **ATP9B** | 4 | XP_855763, XP_855846 ^a^, XP_855968 ^a^, XP_848399 ^a^, XP_855927 ^a^, XP_855885 ^a^ |
| **ATP11A** | 4 | XP_534190 |
| **ATP11B** | 4 | XP_535816 ^c^ |
| **ATP11C** | 4 | XP_538187 |
| **ATP10A** | 4 | XP_545808 |
| **ATP10B** | 4 | XP_546266 |
| **ATP10D** | 4 | XP_859041 ^a,d^, XP_849533 |
| **ATP13A1** | 5 | XP_533862 |
| **ATP13A2** | 5 | - ^h^ |
| **ATP13A3** | 5 | XP_535783 |
| **ATP13A4** | 5 | XP_850465, XP_861466 ^a^ |
| **ATP13A5** | 5 | XP_545252 |

^a^ The sequence has later been withdrawn.

^b^ Now included in NP_001184013.

^c^ Categorized as class 1b by the automatic method. The first 47 amino acids do not align.

^d^ The sequence was not categorized by the automatic method. It is a fragment only including the first 534 amino acids.

^e^ Now covered by XP_003433909.

**Supplementary Table 8.**List of genomes without P-type ATPases found in the genome dataset. # of proteins from the Entrez Genome homepage.

| Genome | # of proteins in genome | Note |
| --- | --- | --- |
| **Archaeal** |  |  |
| *Ignicoccus hospitalis KIN4/I* | 1434 |  |
| *Methanocaldococcus jannaschii DSM 2661* | 1729 |  |
| *Methanopyrus kandleri AV19* | 1687 |  |
| *Nanoarchaeum equitans Kin4-M* | 536 | Only genome sequenced from the Nanoarchaeota Phylum |
| *Nitrosopumilus maritimus SCM1* | 1795 | Only genome sequenced from the Thaumarchaeota Phylum |
| *Pyrococcus horikoshii OT3 ** | 1955 |  |
| *Thermococcus gammatolerans EJ3* | 2156 |  |
| *uncultured methanogenic archaeon RC-I* | 3085 |  |
|  |  |  |
| **Bacterial** |  |  |
| *Anaplasma centrale str. Israel* | 923 |  |
| *Anaplasma marginale str. Florida* | 940 |  |
| *Anaplasma marginale str. St. Maries* | 948 |  |
| *Anaplasma phagocytophilum HZ* | 1264 | No *Anaplasma sp.* have pumps |
| *Bartonella bacilliformis KC583* | 1283 |  |
| *Bartonella grahamii as4aup* | 1737 |  |
| *Bartonella henselae str. Houston-1* | 1488 |  |
| *Bartonella quintana str. Toulouse* | 1142 |  |
| *Bartonella tribocorum CIP 105476* | 2069 | No *Bartonella sp.* have pumps |
| *Baumannia cicadellinicola str. Hc (Homalodisca coagulata)* | 595 | Only genome sequenced from the *Baumannia* genus |
| *Borrelia afzelii PKo* | 850 |  |
| *Borrelia burgdorferi B31 ** | 851 |  |
| *Borrelia burgdorferi ZS7* | 808 |  |
| *Borrelia duttonii Ly* | 820 |  |
| *Borrelia garinii PBi* | 829 |  |
| *Borrelia hermsii DAH* | 819 |  |
| *Borrelia recurrentis A1* | 800 |  |
| *Borrelia turicatae 91E135* | 818 | No *Borrelia sp*. have pumps |
| *Buchnera aphidicola str. 5A (Acyrthosiphon pisum)* | 555 |  |
| *Buchnera aphidicola str. APS (Acyrthosiphon pisum) ** | 564 |  |
| *Buchnera aphidicola str. Bp (Baizongia pistaciae) ** | 504 |  |
| *Buchnera aphidicola str. Cc (Cinara cedri)* | 357 |  |
| *Buchnera aphidicola str. Sg (Schizaphis graminum) ** | 546 |  |
| *Buchnera aphidicola str. Tuc7 (Acyrthosiphon pisum)* | 553 | Only genome sequenced from the *Buchnera* genus |
| *Campylobacter lari RM2100* | 1503 | All other *Campylobacter sp.* have pumps |
| *Candidatus Amoebophilus asiaticus 5a2* | 1334 |  |
| *Candidatus Azobacteroides pseudotrichonymphae genomovar. CFP2* | 758 |  |
| *Candidatus Blochmannia floridanus ** | 583 |  |
| *Candidatus Blochmannia pennsylvanicus str. BPEN* | 610 |  |
| *Candidatus Carsonella ruddii PV* | 182 |  |
| *Candidatus Hodgkinia cicadicola Dsem* | 169 |  |
| *Candidatus Liberibacter asiaticus str. psy62* | 1109 |  |
| *Candidatus Pelagibacter ubique HTCC1062* | 1354 | Some *Candidatus sp.* have pumps |
| *Coxiella burnetii CbuK_Q154* | 1900 | All other *Coxiella sp.* have pumps |
| *Dichelobacter nodosus VCS1703A* | 1280 | Only genome sequenced from a *Dichelobacter sp*. |
| *Ehrlichia canis str. Jake* | 925 |  |
| *Ehrlichia chaffeensis str. Arkansas* | 1105 |  |
| *Ehrlichia ruminantium str. Gardel* | 950 |  |
| *Ehrlichia ruminantium str. Welgevonden* | 958 | No *Ehrlichiqa sp.* have pumps |
| *Francisella tularensis subsp. holarctica FTNF002-00* | 1580 |  |
| *Francisella tularensis subsp. holarctica OSU18* | 1555 | All other *Francisella sp*. have pumps |

* Also reported in PATbase (Axelsen & Palmgren 1998).

Table continued on next page.

**Supplementary Table 8 continued.**

| Genome | # of proteins in genome | Note |
| --- | --- | --- |
| **Bacterial continued** |  |  |
| *Haemophilus ducreyi 35000HP ** | 1717 |  |
| *Haemophilus somnus 129PT* | 1792 | All other *Haemophilus sp*. have pumps |
| *Helicobacter mustelae 12198* | 1400 | All other *Helicobacter sp.* have pumps |
| *Mycoplasma gallisepticum str. R(low)* | 763 |  |
| *Mycoplasma mobile 163K* | 633 | All other *Mycoplasma sp.* have pumps |
| *Neorickettsia risticii str. Illinois* | 892 |  |
| *Neorickettsia sennetsu str. Miyayama* | 932 | No *Neorickettsia sp.* have pumps |
| *Orientia tsutsugamushi str. Boryong* | 1182 |  |
| *Orientia tsutsugamushi str. Ikeda* | 1967 | Only genome sequenced from the *Orientia* genus |
| *Rickettsia africae ESF-5* | 1030 |  |
| *Rickettsia akari str. Hartford* | 1258 |  |
| *Rickettsia bellii OSU 85-389* | 1475 |  |
| *Rickettsia bellii RML369-C* | 1429 |  |
| *Rickettsia canadensis str. McKiel* | 1090 |  |
| *Rickettsia conorii str. Malish 7 ** | 1374 |  |
| *Rickettsia felis URRWXCal2* | 1400 |  |
| *Rickettsia massiliae MTU5* | 968 |  |
| *Rickettsia peacockii str. Rustic* | 927 |  |
| *Rickettsia prowazekii str. Madrid E ** | 835 |  |
| *Rickettsia rickettsii str. 'Sheila Smith'* | 1343 |  |
| *Rickettsia rickettsii str. Iowa* | 1383 |  |
| *Rickettsia typhi str. Wilmington* | 838 | No *Rickettsia sp*. have pumps |
| *Wigglesworthia glossinidia endosymbiont of Glossina brevipalpis ** | 611 | Only genome sequenced from the *Wigglesworthia* genus |
| *Wolbachia endosymbiont of Culex quinquefasciatus Pel* | 1275 |  |
| *Wolbachia endosymbiont of Drosophila melanogaster* | 1195 |  |
| *Wolbachia endosymbiont strain TRS of Brugia malayi* | 805 |  |
| *Wolbachia sp. wRi* | 1150 | No *Wolbachia sp.* have pumps |
| *Xylella fastidiosa 9a5c ** | 2766 |  |
| *Xylella fastidiosa M12* | 2104 |  |
| *Xylella fastidiosa M23* | 2161 |  |
| *Xylella fastidiosa Temecula1 ** | 2034 | Only genome sequenced from the *Xylella* genus |

* Also reported in PATbase (Axelsen & Palmgren 1998).

**Supplementary Table 9.** The 217 UniprotKB sequences placed in Class 0.

| **Domain** | **UniprotKB accession number** |
| --- | --- |
| **Eukaryota** | A2RBM0, A4QZI1, A4R2M7, A6RRE4, A7EMW9, A8NB79, A8NX80, B2AR58, B7XIA3, B7XIM2, C4V9V6, C4YL00, C6H2M2, Q0TXF1, Q0UDG4, Q1K6I5, Q2GSR4, Q4P4C5, Q5A9B3, Q5A9L4, Q703G4, Q8SRH4, A0BYG9, A0C2C2, A0CTE9, A0DB25, A0DKE2, A0E0W6, A0E3A3, A0E3K1, A0E4U6, A2E555, A2FJ70, A3FKK1, A8BV45, A8NZD8, A8VUU1, A8VUX1, A8VUX6, A8VUY0, A8VUY5, A9V756, B6C806, B6C814, B6C817, B6C821, B6C822, B6C826, B6C829, B6C831, B6C833, B6C834, B6C836, B6C839, B6C843, B6C845, B6K8Q8, B9PK54, C4QTL6, C5KCN8, C5KDA8, C5L4N0, C5LBB0, C5LF82, C5LS84, C6LMT0, C6YYS4, C6YYS5, C6YYS6, C6YYS7, C6YYS9, C6YYT2, C6YYT3, C6YYT4, C6YYT5, C6YYT6, C6YYT7, C6YYT8, C6YYU1, C6YYU2, C6YYU3, C6YYU4, C6YYV0, C6YYV1, C6YYV5, C6YYV8, Q23RI2, Q27851, Q27852, Q3SDA8, Q3SDA9, Q3SDB0, Q3SDB2, Q3SEE2, Q3SEE4, Q3SEE7, Q3SEE8, Q3SEF3, Q4XRL5, Q5CFJ7, Q5CIC5, Q5CT66, Q5CTJ9, Q5CYZ4, Q6RXX1, Q95049, Q9U5I3, Q9XZJ9, Q9Y174, Q9Y175, Q9Y176, A4S8G9, A5B2F3, A7QSX3, A7R0H6, A7R8M2, A9T9F0, B8BVH9, B9H6P8, B9T5P6, Q00U35, B3SHZ8, B3SHZ9, B3SI00, B5A4T0, Q5IR98, Q5IRA2, Q8UWA2 |
| **Bacteria** | Q8Z8E5, Q8R8I6, A0YJM6, A0YKE2, A3IYD8, A3YW50, A3Z8I9, A6BL04, A6PRQ0, A7B356, A7JEV4, A7JJV4, A7JP17, A7YSC8, B0MLP7, B1B8G9, B4UYI9, B5USU2, B5W284, B5WEQ3, B9YJ05, C0DAR0, C0EIQ0, C0WNM0, C0XJV9, C0YHF4, C1IC41, C2CZR3, C2E0U6, C2EQK6, C2GUC9, C2HM14, C2L0C2, C2XQP8, C3XKH2, C4G0U7, C4VLQ7, C5E7S6, C5F273, C5RLN7, C5TB29, C5VNN0, C5ZWG8, C6I016, C6N6M8, C6PH47, C6PL70, C6YTP3, Q0EYI5, Q83VA0, Q9L721, A0LLJ5, A0Q357, A4IVV8, A5IB17, A6M1V3, A8AYM3, A8YX51, A9F987, B0SVN9, B0TWK1, B1WZD4, B1XIA7, B2ITY3, B2SEB3, B3DSW4, B3E056, C1DHA2, Q10VG7, Q183R9, Q2IPD6, Q5WXG7, Q5X643, Q5ZSY5, Q5ZWF9, Q6ABJ1, Q72RN5, Q8F426, Q8G5H5 |
| **Archaea** | A0B7P4, A0B7X0, A2SS48, B8D5Z6, B8GJD8, O26581, Q12XJ2, Q2FN38 |
| **Virus** | A7IUR5, A7RCN8 |


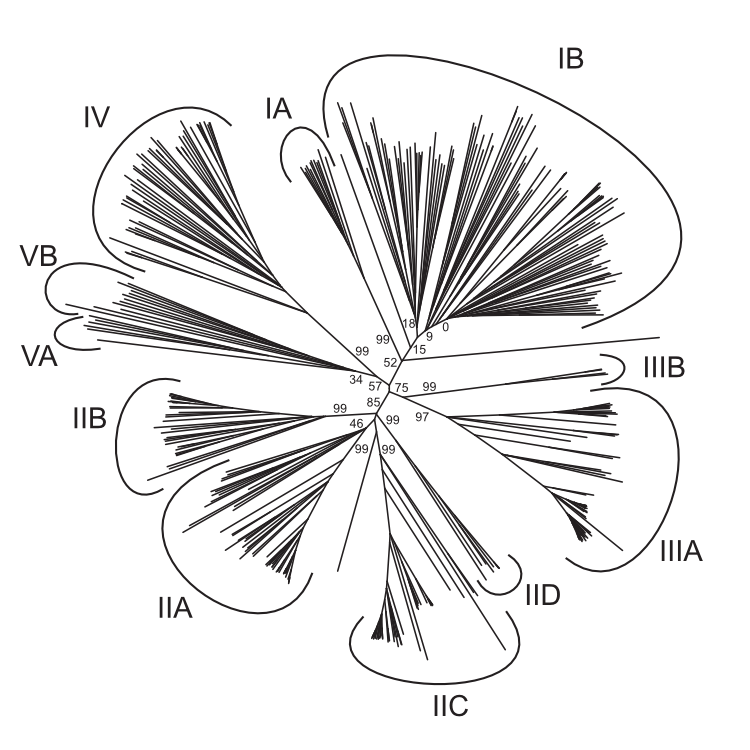


**Supplementary Figure 1.** Minimum evolution tree of the sequences found in PATbase (http://www.traplabs.dk/patbase). Sequences in PATbase were aligned using MUSCLE, and a minimum evolution tree build in MEGA4. The numbers denote bootstrapping values from 500 trials.


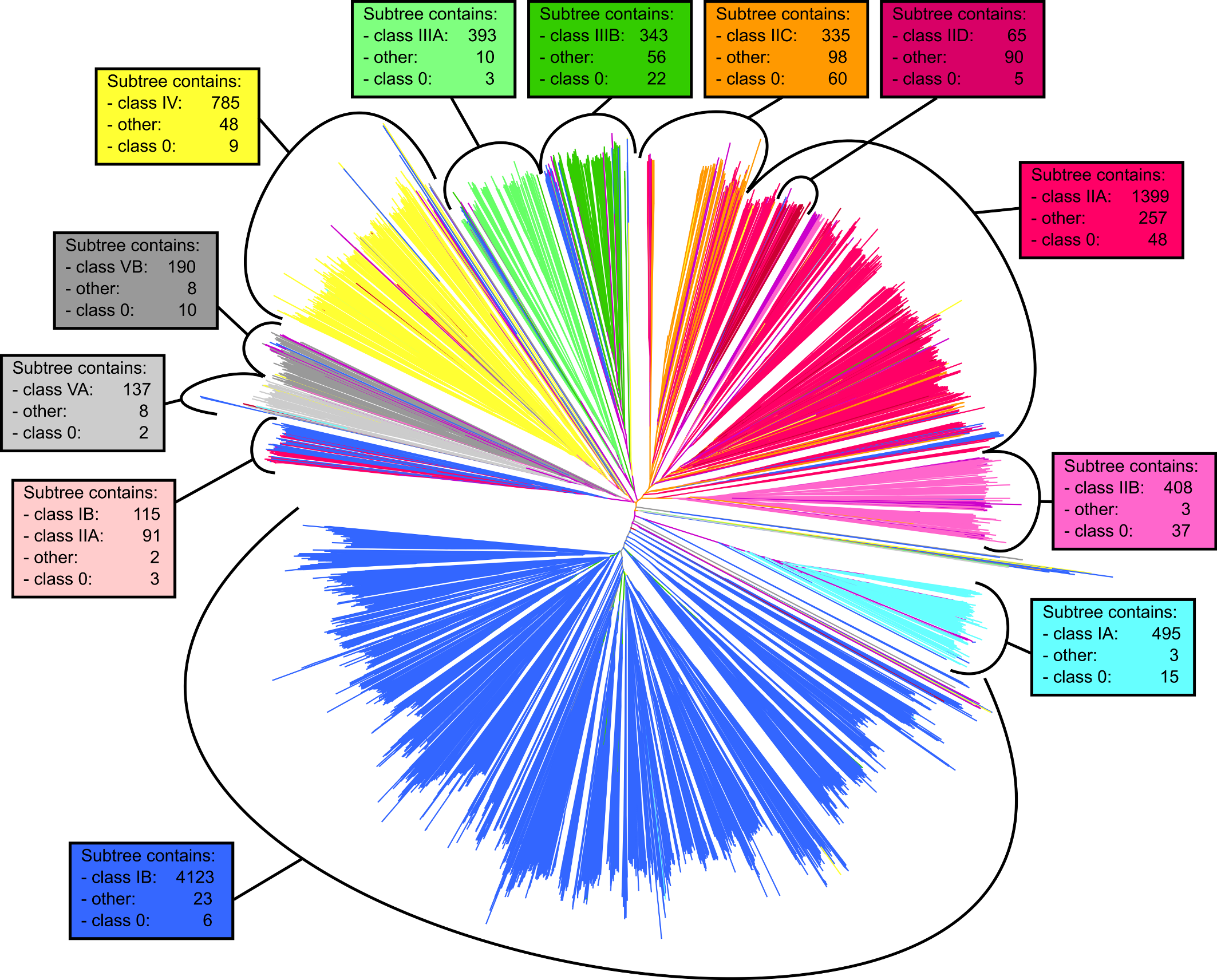


**Supplementary Figure 2.** Neighbour-joining tree of all sequences identified as P-type ATPases by the SLR-classifier in Task 1. 12 subtrees are highlighted, of which 11 correlates with canonical classes found by SLR. The boxes show the SLR-distribution found within subtrees and the branches are colored according to the SLR classification.


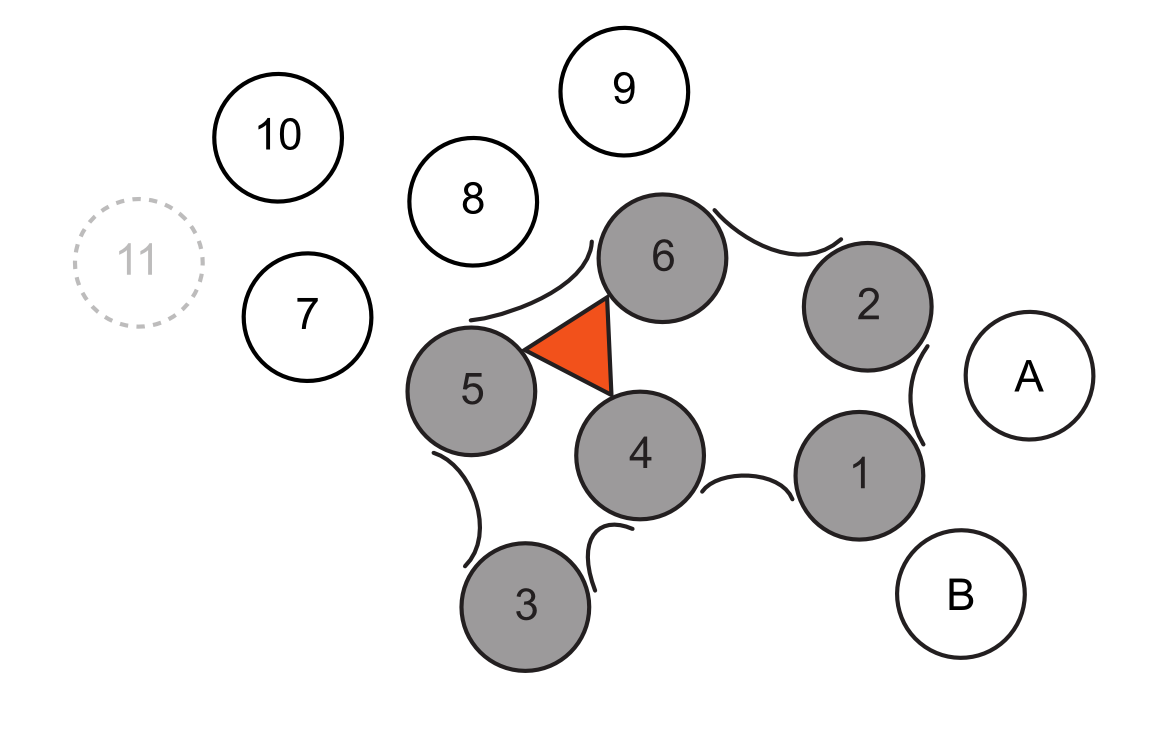


**Supplementary Figure 3.** Suggested membrane topology in 'space fill' viewed perpendicular on the membrane plane, based on known structures of P-type ATPases, as well as sequence alignments and biochemical studies (e.g. [4-6,34,37-39]). The figure shows the core element as well as the C-terminal element (7-10 here) and the N-terminal element (A-B here). The position of a possible 11th helix in the C-terminal element is shown with a dotted circle overlapping with the position of the gamma subunit in Na^+^/K^+^-ATPase [4,34]. The orange triangle denotes the cation binding site in Ca^2+^-ATPase and Na^+^,K^+^-ATPase and is conserved throughout the family based on sequence alignments and mutational studies on various P-type ATPases.


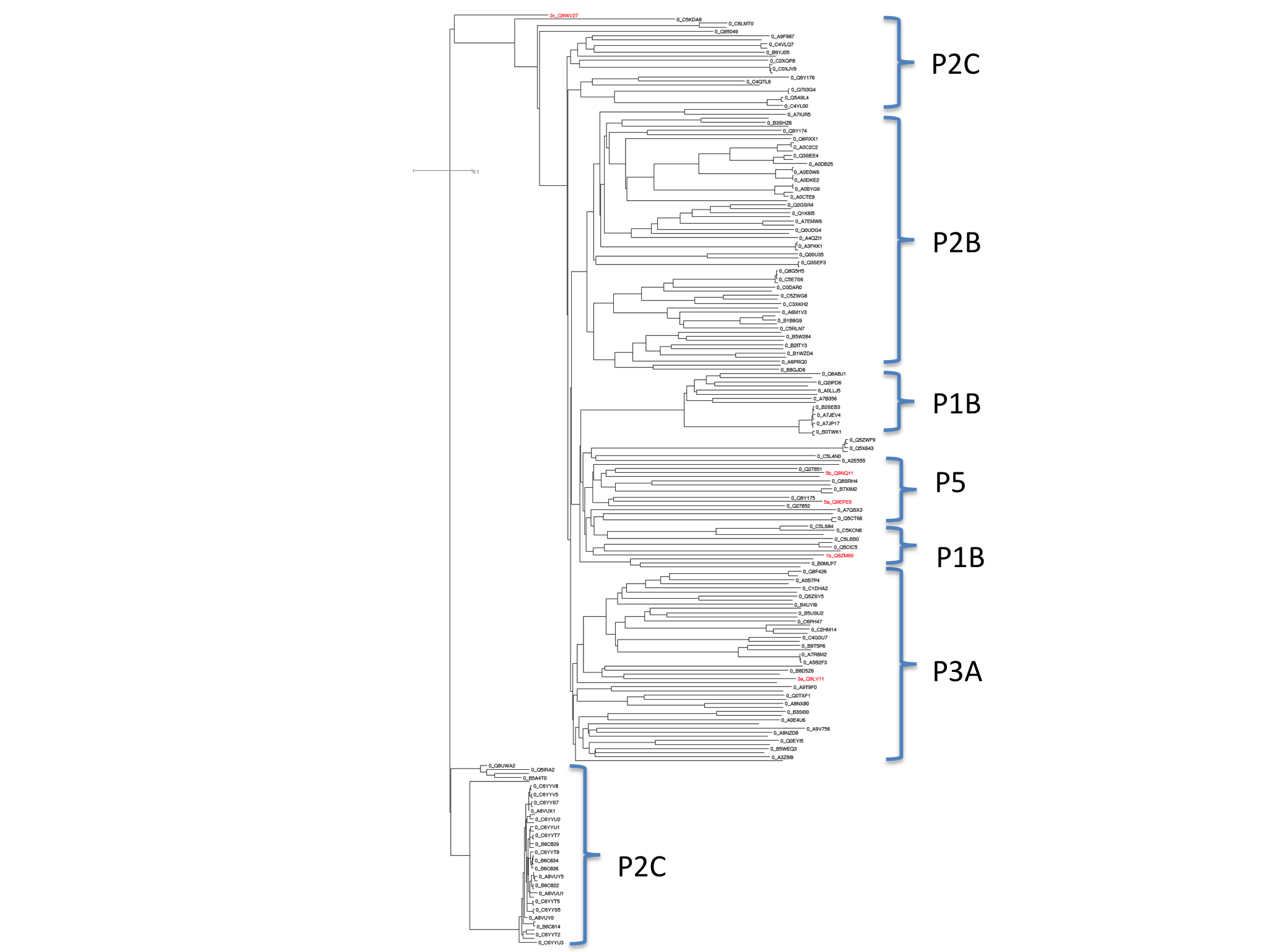


**Supplementary Figure 4.** Neighbour-joining tree of the 217 unclassified P-Type ATPases by our SLR-classifiers (Task 2). All of these 217 sequences are P-Type ATPases and a manual inspection of these reveals their type as shown by the annotation of the tree. It shows that some subfamilies more often than others are misclassified.


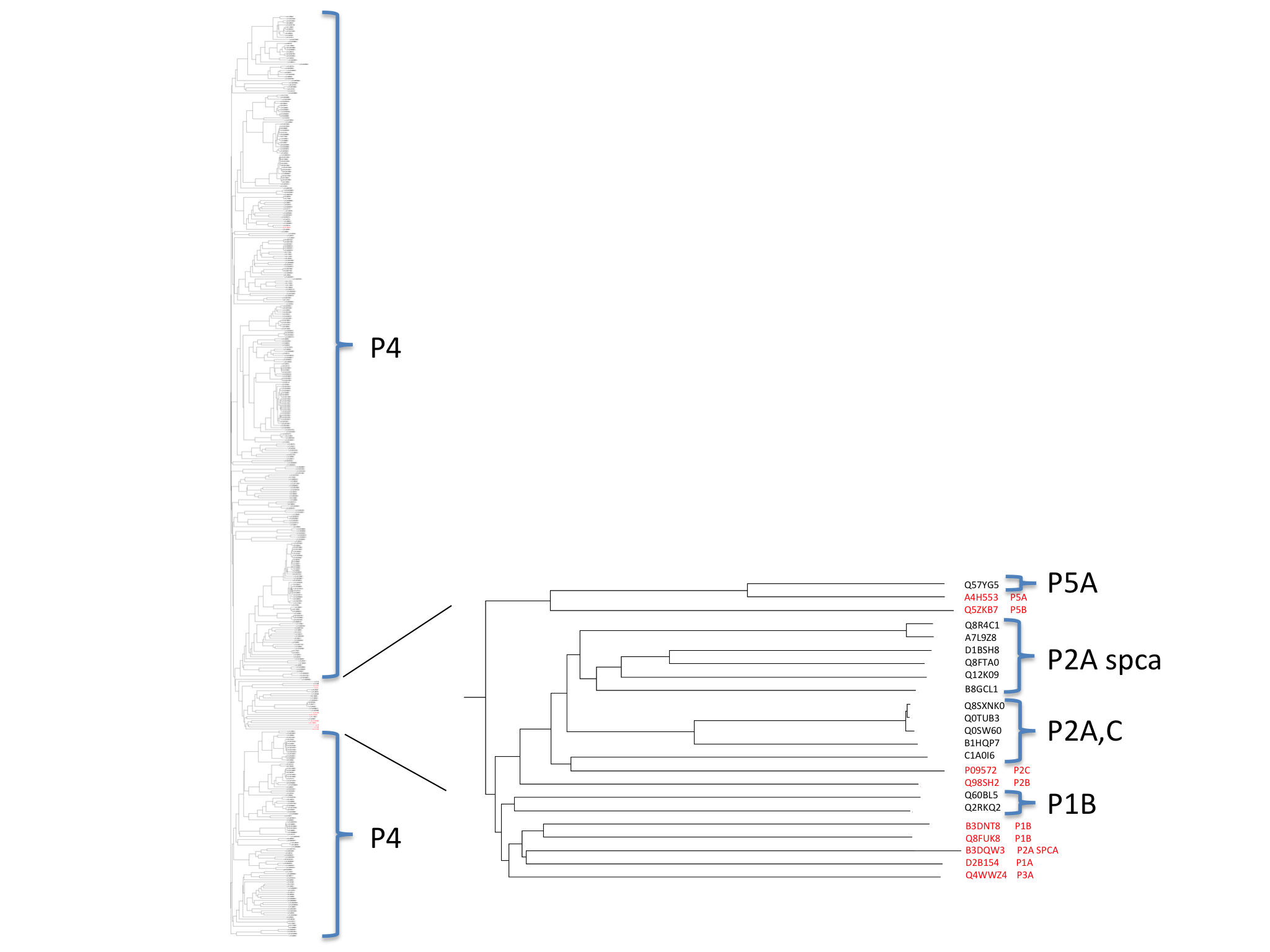


**Supplementary Figure 5.** Neighbour-Joining tree of the 840 P-Type ATPases classified as Class IV by our SLR-classifiers (Task 2). It shows that 23 of these sequences are in a subtree together with outgroups of other P-Type ATPase subfamiles.
